# The Quantum Brain: The Untold Story of Docosahexaenoic Acid’s Role in Brain Evolution, Biophysics, and Cognition

**DOI:** 10.3390/ijms262311542

**Published:** 2025-11-28

**Authors:** Michael A. Crawford, Lawrence A. Horn, Thomas Brenna, Catherine Leigh Broadhurst, Simon C. Dyall, Mark Johnson, Walter F. Schmidt, Andrew J. Sinclair, Manahel Thabet, Yiqun Wang

**Affiliations:** 1 Institute of Brain Chemistry and Human Nutrition, Imperial College, London SW7 2AZ, UK; mark.johnson@imperial.ac.uk (M.J.); yiqun.wang@imperial.ac.uk (Y.W.); 2 Independent Researcher, Bethesda, MD 20816, USA; horn.larry@gmail.com; 3 Dell Pediatric Research Institute, Dell Medical School, Austin, TX 78712, USA; tbrenna@gmail.com; 4 US Department of Agriculture Agricultural Research Service, Beltsville, MD 20705, USA; leighbroadhurst@verizon.net (C.L.B.); walter.schmidt4870@gmail.com (W.F.S.); 5 School of Life and Health Sciences, University of Roehampton, London SW15 5PU, UK; simon.dyall@roehampton.ac.uk; 6 Faculty of Health, Deakin University, 221 Burwood Highway, Burwood VIC 3125, Australia; andrew.sinclair@deakin.edu.au; 7 King Abdulaziz City for Science and Technology (KACST), P.O. Box 11442, Riyad 11442, Saudi Arabia; manahel@manahelthabet.com

**Keywords:** chromophore, docosahexaenoic acid (DHA), electron tunneling, entanglement, Hamiltonian, Hebbian Learning, Hilbert, methylene interruption, semiconductor

## Abstract

Docosahexaenoic acid (DHA), the dominant polyunsaturated fatty acid in photoreceptors, neurons, and synapses, is usually described as a passive structural membrane constituent. We propose a different view: DHA is a quantum-electronically active molecule whose methylene interrupted double-bond system creates an electron-rich matrix that couples with proteins to form quantum “clouds” and high-speed signaling central to recognition, recall, and cognition. Integrating evidence from molecular evolution, biophysics, and neuroscience, we argue that, as the original chromophore, DHA’s unique properties enabled the emergence of the nervous system and continue to provide the electronic substrate for cognition. By suggesting that cognition depends not only on protein-based mechanisms but on DHA-mediated electron dynamics at the membrane–protein interface, this perspective reframes DHA as an active, conserved determinant of brain evolution and function.

## 1. Introduction

The search for a physical basis of cognition has often centered on proteins, genes, and synaptic networks. Yet these components rest upon a molecular foundation that has received far less attention: the lipid composition of neural membranes. Among these lipids, docosahexaenoic acid (DHA, 22:6n-3) constitutes a major fraction of brain and retinal membranes. This is no accident. DHA’s six methylene interrupted double bonds endow it with quantum properties enabling delocalization of electrons at a precise energy level essential for neural information transfer. 

Traditional models cast DHA as a structural lipid ensuring membrane fluidity, but this is inadequate to explain its strict conservation across 600 million years of nervous system evolution. We advance the perspective that DHA’s electronic properties, derived from its six methylene-interrupted double bonds, position it as the quantum foundation of cognition.

Evolutionary evidence underscores the centrality of DHA. From the Cambrian explosion onward, proteins and genes have diversified, but DHA and other brain lipids have remained compositionally unchanged, suggesting constraint on the design of nervous systems—an invariant “molecular fossil” whose physical properties shaped neural architectures.

Recent insights into membrane biophysics and quantum biology now provide a lens for reconsideration. Building on our earlier work regarding DHA’s structure, its extreme conservation in electrical signaling membranes of the brain, and its role in visual transduction, we advance here an explanation of why DHA’s polyunsaturated structure permits electron tunneling, exciton migration, and resonance energy transfer within the lipid bilayer. These quantum phenomena may be amplified in the densely packed and highly ordered environment of synaptic membranes. Rather than acting as passive scaffolds, DHA-rich domains can be viewed as active participants in information processing.

Here, we integrate molecular evolution, quantum biology, and membrane dynamics into a unified framework: (1) DHA as a primordial chromophore behind the origin of nervous systems; (2) the electrochemical and quantum properties of DHA in neural membranes; and (3) the role of DHA-mediated electron dynamics in recognition, recall, and plasticity. Together, these considerations point to a new paradigm in which the brain, being largely made of lipids, is understood as a lipid–electron system with DHA at its core.

## 2. Results and Discussion

As discussed below, our hypothesis regarding DHA’s role in electrical signaling, rooted in quantum biology and membrane dynamics, provides the only plausible explanation for the extraordinary speed and precision required by the memory, recall, recognition, and cognition capabilities of the human brain. No other theory can explain how Mozart could have written the entire score from “Miserere mei, Deus (Have mercy on me, O God)” by Gregorio Allegri from memory at age 14 after hearing it for the first time or composed the piano concerto no. 7 for orchestra and two pianos (KV 242) consisting of 15,540 notes split between four sets of five fingers without any future need of correction, and how audiences would witness the speed, precise synchronization, recall, execution, and coordination between two individuals and orchestra required to perform it; how Jon Yu Jong took just nine seconds to look through 52 randomly shuffled playing cards and memorize their order at the 2019 World Memory Championship; or how vocal electrodynamics can connect with the sound of each word in the poem “To Daffodils” by Robert Herrick and bring to mind the image of their brief radiance with the words “Fair daffodils we weep to see, you haste away so soon”.

DHA’s unique biochemical and biophysical properties, preserved through 600 million years of evolution, made it the original chromophore, conferring excitonic transfer and rapid signaling advantages critical to the evolution of the nervous system with a quantum-optimized environment for information integration to become the molecular foundation of recognition, recall, and neural dynamics. Conventional accounts of memory formation that emphasize the strengthening of synaptic protein density through repetitive sensory formulation, being too slow to operate at the speed required, cannot account for this phenomenon. Radical though it may sound, the lipid–electron ensemble enabled by DHA is the only explanation. We shall take you step-by-step through our analysis.

### 2.1. DHA as a Primordial Chromophore

**Figure 1:**
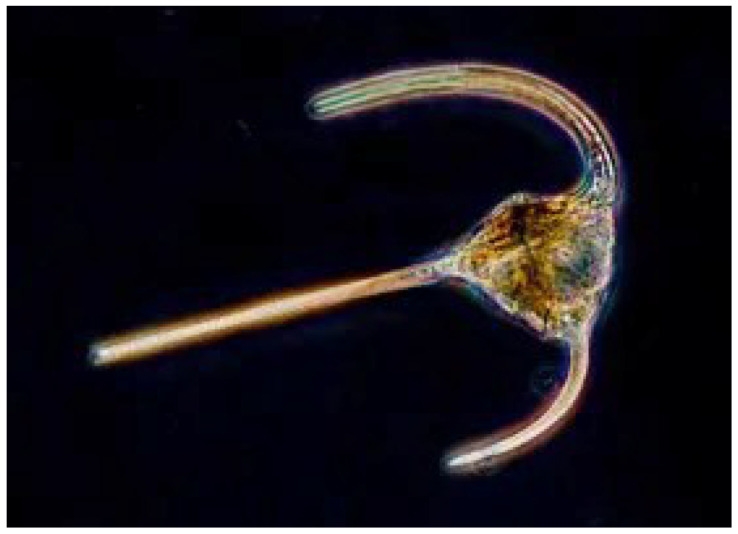
Dinoflagellate. (*Encyclopædia Britannica* ©blickwinkel/Alamy) DHA has served the construction of visual cell membranes, neurons, and synapses throughout the evolution of multicellular systems, starting with the dinoflagellate.

**Figure 2:**
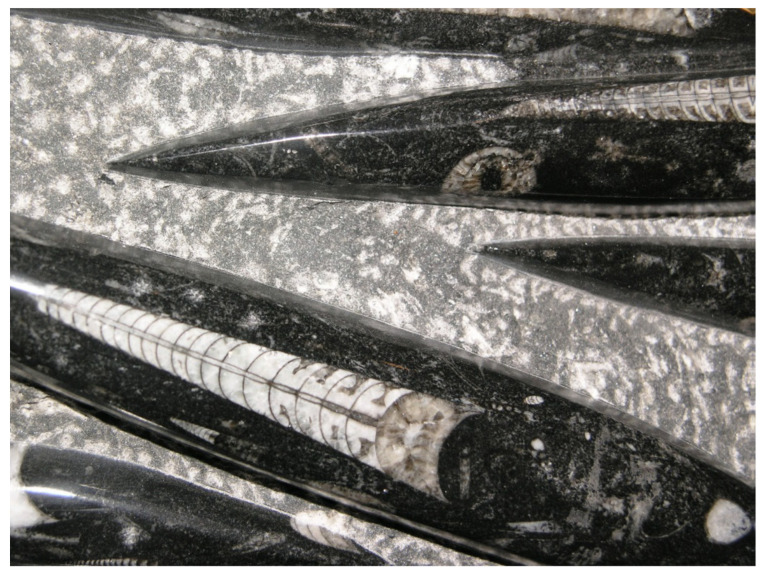
Orthoceras. (Photo of fossil held by MAC, origin: Morocco.) Prehistoric cephalopod 488–443 m.y.a., a possible precursor of squids. The eye looks similar to the squid eye, which in turn bears a similarity to the human eye.

The “conditions of existence” [1] leading to vision and the brain were oxygen and the absence of an ozone layer, leaving the planet bathed in high-energy UV solar radiation. The dinoflagellate (Figure 1 below) has an eye spot that can both photosynthesize and see. It is rich in DHA and contains Di-DHA phosphoglycerides present in eyes today [2]. DHA would have absorbed UV, thereby activating a π-electron into escape mode. The extrusion of electrons would activate movement by taking the cells toward the light at the surface where there was food. (See also Figure 2 below).

### 2.2. Both Retinal and DHA Are Highly Unsaturated Molecules

#### 2.2.1. Conjugated Double Bonds: R1-CH=CH-CH=CH-CH=CH-CH=CH-R2

In retinal molecules, the double bonds in the side chain are in the TRANS configuration; except for the 11-cis, the photon acceptor is conjugated, which means the electrons are free to move along the molecule. DHA’s unique six methylene-interrupted double bond geometry resembling chromophores in photoreceptive proteins, however, are in the CIS configuration, which is at a higher energy state than TRANS, and the -CH2- between the double bonds restricts the movement of the π-electrons to their respective wells. 

In methylene interruption, more energy is required to release an electron than with conjugation. This makes DHA a resistor [3].

#### 2.2.2. Methylene Interruption: R1-CH=CH-CH2-CH=CH-CH2-CH=CH-CH2-CH=CH-CH-R2

The confinement of the π-electrons to their wells by methylene interruption would have made the molecule more stable in the UV-bathed conditions of the Ediacaran and Early Cambrian periods. By contrast with retinal molecules, DHA’s methylene interruption ensures that more energy is required to excite an electron from a CIS double bond, as reflected by its peak of absorption in the middle of the solar UV range.

**Figure 3:**
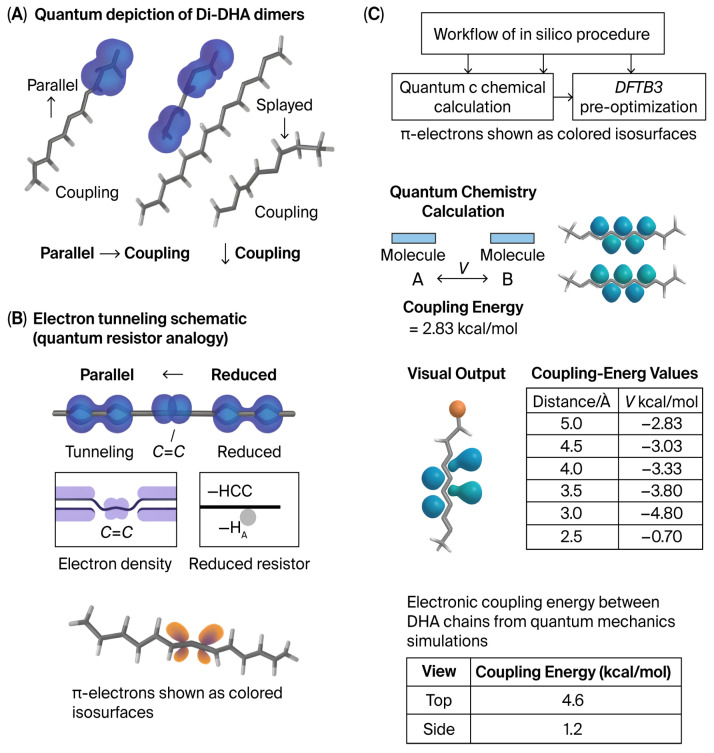
Di-DHA orientation and π-electron localization. (MD-supplied geometries only; all electronic data are quantum-mechanical) (**A**) Two representative Di-DHA dimers—parallel (**left**) and splayed (**right**)—were extracted from classical molecular dynamics (MD) and analyzed by quantum chemistry (DFTB3 pre-optimization ωB97X-D/def2-SVP, PCM ε ≈ 2); both single-point and time-dependent methods were used to visualize electron-density difference maps and evaluate electronic couplings (H_AB) through the generalized Mulliken–Hush (GMH) method and transition–density couplings via Time-Dependent Density Functional Theory (TDDFT). (**B**) Colored isosurfaces depict π-electron density differences across cis C=C bonds, illustrating regions of highest electron probability. (**C**) With Di-DHA phosphoglycerides, parallelism is possible as shown. Parallel configurations display greater π-density overlap and stronger inter-fragment electronic coupling (H AB) consistent with lower effective resistance to charge or exciton transfer. In two adjacent phosphoglycerides, finding a low thermodynamic state in parallel with a reduction in resistance, MD shows the π-electrons lying closer together than they do on the chain [4,5,6].

Two resistors in series result in the summation of the resistance:

Resistances in Series   Viz   5 Ω + 15 Ω, = 20. Ω,

However, if two resistors are in parallel, then the resistance drops:

Resistances in Parallel Viz 10 Ω ⟷ 10 Ω, = 5. Ω,

With DHA being a planar molecule, two DHAs in parallel as in Di-DHA would reduce the resistance, thereby facilitating the flow of an electrical current in low light for the earliest air-breathing life (see Figure 3 below). This property of DHA transcends its traditional role in energy metabolism, positing it as a critical factor in refining synaptic signaling fidelity.

**Figure 4:**
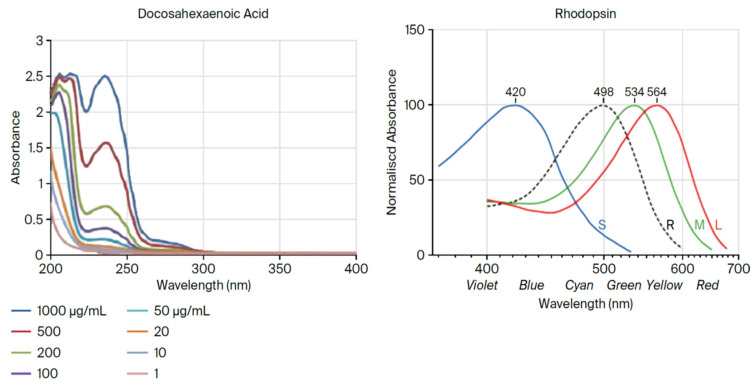
Light absorption curves of DHA and rhodopsin [8].

As the ozone layer formed, Nature had to find a new way to convert energy from the sun. During extant photoreception, an 11-cis electron in retinal absorbs the energy of a photon as described above. However, today DHA remains in attendance surrounding rhodopsin to enable the final step in the high-speed transduction of electron wave function [7]. (See Figure 4 below).

**Figure 5:**
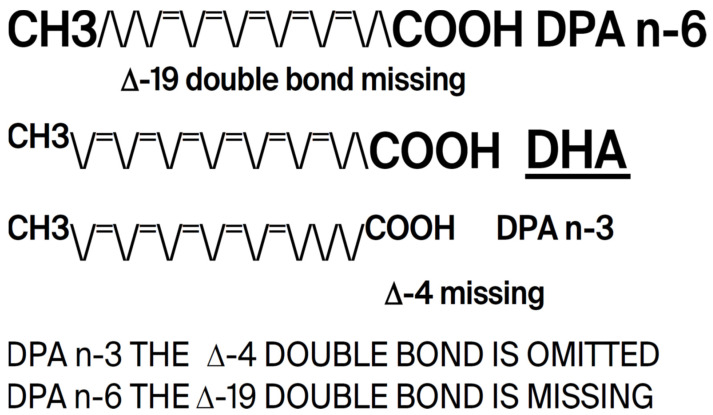
DHA, the overlord of DNA (constructed using Word and PDF).

The conservation of DHA is striking. In 600 million years of evolution, n-3 DPA (Docosapentaenoic acid), a precursor of DHA, is present only in small amounts in the brain where the major n-3 fatty acid is consistently found to be DHA [9,10]. But despite being easier to synthesize, less susceptible to peroxidation in an oxygen-fueled locality, and more readily available especially in large herbivorous mammals, DPA never replaced DHA. Rather, DHA has served the construction of visual cell membranes, neurons, and synapses throughout the evolution of multicellular systems, and starting with the dinoflagellates, its presence is found in high proportion in the photoreceptors and neural membranes of its descendants including orthoceras, cephalopods, fish, amphibia, reptiles, birds, mammals, and primates. The principal difference between DHA and DPA is that DPA has only one double bond, and as evidence of its criticality, DHA has two. (see Figure 5 below). As such, DHA in some sense may be considered the overlord of DNA.

**Figure 6:**
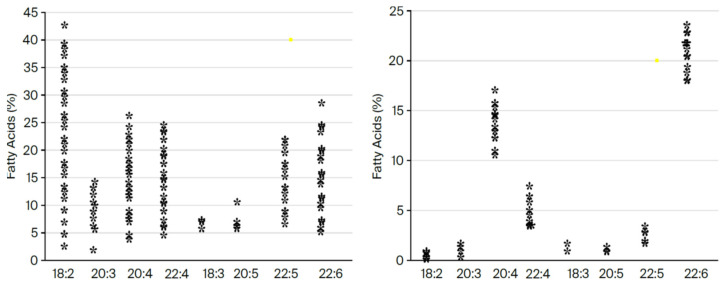
Variation in EPG fatty acids in liver and brain in 36 mammalian species. (Constructed by MAC using Word.) Shown here are the variation in the fatty acid composition of the ethanolamine phosphoglycerides from the liver (plotted on the **left**) compared with the minimal variation in samples from the motor cortex (plotted on the **right**) from our original analysis of 32 species [13], which later expanded to 42 species and then included the dolphin and gray whale [2,14]. This consistency in natural selection to choose DHA in preference to two identical molecules, except for missing one double bond, is meaningful in chemical, physical, and biological function [15,16,17,18].

Neuronal and synaptic membranes are enriched in DHA, a feature not shared by astrocytes or the vascular endothelium which prioritize arachidonic acid [11,12]. While other fatty acids vary between species and diets, DHA is maintained with minimal alteration, suggesting that once it is integrated into membranes, DHA’s properties determined excitability, photoreception, and synaptic signaling (See Figure 6 below).

Lipid biosynthesis is twice as expensive energetically compared to carbohydrate or protein synthesis. Hence, there is no intracellular detail seen in the fossil record for the first three billion years of anaerobic life, but with the availability of oxygen, the biological synthesis of lipids and cell membranes became more feasible. With heightened UV energy input leading to the biological synthesis of membrane lipids present in dinoflagellate (or similar) lipids, we propose that DHA was the original chromophore for photon transduction to electrons, conferring excitonic transfer and rapid signaling advantages with minimal energy loss critical to the evolution of the nervous system with a quantum-optimized environment for information integration [19]. 

As evidenced in the fossil record, the natural emergence of lipid membranes [20] with their ability of self-assembly [21] would have led to intracellular specialization, cell specialization, and the multicellular life associated with air-breathing organisms [14,22]. In the detail are lipid bi-layers, which, in conjunction with the evolution of lipophilic proteins, form the nuclear envelope, the microsomes, mitochondria, reticulo-endothelium, and plasma membrane [23].

As life became multicellular, the generation of electro-magnetic pulses would have bred lipid pathways and, hence, a nervous system and ultimately a central nervous system that is the brain. In addition to the evidence that electrons can encourage chemical synthesis [19], Michael Faraday’s first law of electrolysis describes the attraction of a substance to an electrode:

m/Q = Z

where the mass (m) of a substance deposited or liberated at an electrode is directly proportional to the charge Q (in ampere seconds or coulombs); Z, the constant of proportionality, is called the electro-chemical equivalent (ECE) of the substance. Thus, the ECE can be defined as the mass of the substance deposited or liberated per unit charge. 

**Figure 7:**
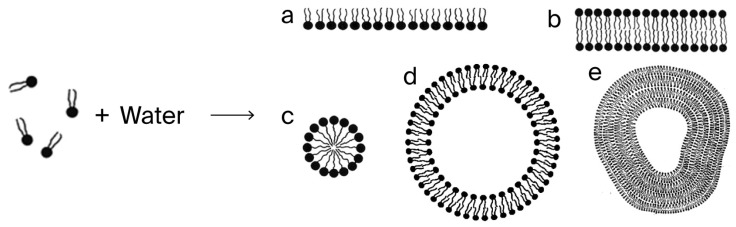
Lipids in water spontaneously form cell-like structures (Illustration by courtesy of Ole G. Mouritsen). Letters denote sequence.

**Figure 8:**
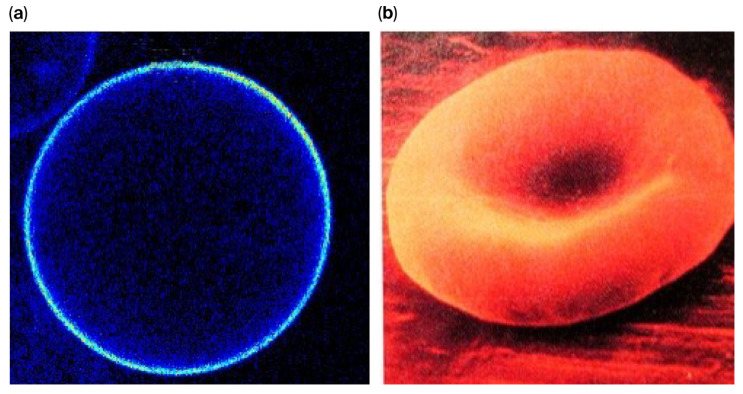
(**a**) Micelle (**b**) Red cell (with thanks to Professor Ole Mouritsen, Department of Food Science, University of Copenhagen).

**Figure 9:**
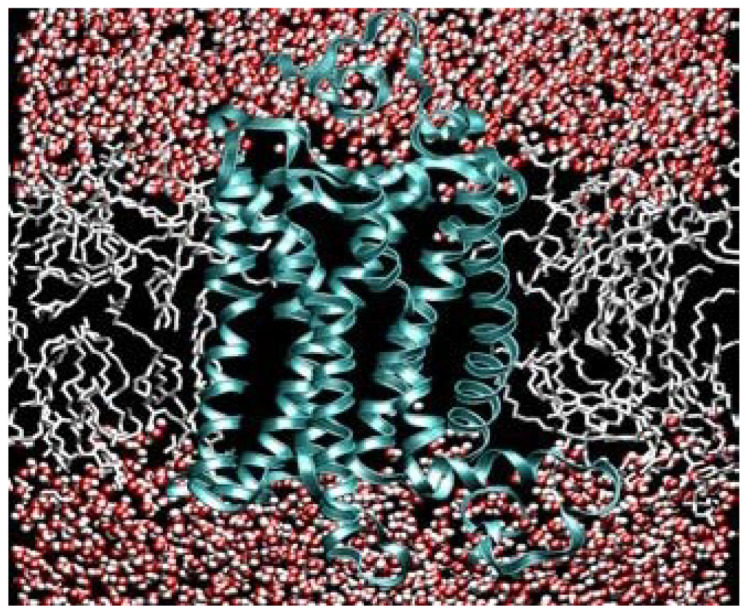
Illustration of a lipid bilayer with a transmembrane protein (with thanks to Klaus Gawrisch, National Institutes of Health). The red and white dots are water molecules, each with its own dipole: red for hydrogen and white for the negativity of the oxygen.

When the Pasteur Point was breached for oxygen tension, facilitating aerobic metabolism, the UV-heightened energy input would have made possible the biological synthesis of lipids, and the lipids would spontaneously form micelles, leading to their use in the formation of cell membranes (see Figure 7, Figure 8 and Figure 9 below).

### 2.3. Electrochemistry and Quantum Properties of DHA

**Figure 10:**
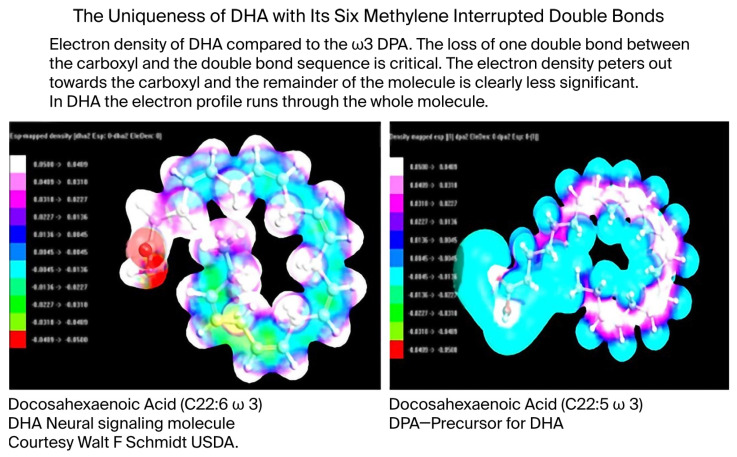
The uniqueness of DHA (calculated and visualized by commercial computational chemistry, HyperChem 8.0.10 for Windows. Molecular Modeling System Updated 2011, Hypercube, Inc. Gainesville, FL 32601 USA). Comparison of the electron density of DHA and its n-3 DPA precursor with one less double bond is shown here. The scale is normalized differential electronegativity. The result shown illustrates electron density across the whole of DHA, which is not the case with DPA.

Beyond its evolutionary role, DHA exhibits unique electrochemical behaviors. The six double bonds create a delocalized π-electron system that supports rapid electron tunneling and excitation migration. Unlike saturated or monosaturated chains, DHA reduces activation barriers, enabling membranes rich in DHA to function as electron-conducting lattices—a property rare among biological lipids. (See Figure 10 below). 

At the nanoscale, DHA may facilitate quantum tunneling of electrons between proteins embedded in the membrane. Tunneling probabilities increase exponentially with decreasing barrier width, and DHA’s flexible chains can transiently reduce distances between donor and acceptor sites. In addition, exciton migration may occur along DHA arrays, providing a mechanism for ultrafast energy transfer. Similarly, neural membranes densely packed with DHA and proteins may provide conditions for sustained coherence and resonance energy transfer.

Such processes are not merely theoretical. Challenging long-held assumptions that warm, noisy environments preclude quantum effects, quantum biological coherence and entanglement have been demonstrated in photosynthetic complexes at biological temperatures [24]. With the benefit of DHA, the brain is capable of remarkable things.

### 2.4. Recognition, Recall, and Neural Dynamics

Conventional accounts of memory formation emphasize the strengthening of synaptic protein density through repetitive sensory formulation, but just as George Wald’s concern with ion and protein functions in photoreception after the adoption of rhodopsin being too slow to explain visual reception [25], the generation and execution of ion movements or protein synthesis in synapses or neurons are too slow to operate at the speed required to initiate motor function in recall and cognition traditionally ascribed to synaptic plasticity. 

This raises the likelihood that neural signaling involves more than ionic flux and neurotransmitter diffusion. Quantum entanglement and coherence—mediated by DHA’s unique electronic structure—may support integration across neural networks at speeds and efficiencies beyond classical models [26]. This would explain rapid visual processing, binding of distributed sensory inputs, and even the neural correlates of consciousness. 

Each of the dendrites that emerge from a neuron has up to 10,000 synapses per cell, totaling some 150 trillion synapses [27,28]. Synapses are in line along the dendrites; hence, a signal from a nerve cell has to travel past one, then another, and another. It is estimated that there are at least 10^11^ (100 billion) neurons times an average of 7000 dendrites or 7.0 × 10^4^ connections which is 7.0 × 10^15^ (7,000,000,000,000,000) or 7 quintillion connections. Each neuron operates once every 10 ms or 100 times per second, or 700 × 10^15^ or 700 quintillion operations per second. This assessment represents a large repository with a high speed of multiple functions. It is further established that “the chemical connections between neurons are effectively “analog”, or floating-point, so the correct term isn’t “bits per second” but FLOPS^A^”. Collectively, our estimate is 700 exaFLOP^vi^ per second. By comparison, the new supercomputer being installed at Argonne Labs at the time of writing operates at 1 exaFLOPS. 

We propose a hypothesis similar to David Marr’s concept of a neural model of the outside world [29], i.e., that the synapses themselves, not the neurons, are storing models of the real world that constitute our memories in an identifiable geography of electro-magnetic, quantum clouds.

The synapses are under the influence of the resting membrane potential, which is the potential between the interior and exterior of the cell of about 80 to 70 millivolt. Therefore, neural networks can be in a superposition of the state “firing” and “resting”. This concept matches the long-established principle of neuronal action potentials being generated by ionic movements across cellular membranes through changes in protein channels created by the opening and closing of ion gates, with sodium ions moving into and potassium ions moving out of the cell. That creates an electronic difference across the cell membrane, causing the conduction to jump from node to node. With the fastest conduction velocity being 80–120 m/s [30], none of this classical description is remotely fast enough to explain information processing in the brain, which is in the milli-second range and plausibly much faster.

David Marr has proposed that memories are made up of models which need markers in specific locations to be recognized as individual identities in a cloud of synapses [29]. Looking at a face results in a flood of electromagnetic waves descending from the two retinas, with multiple wave functions decoded by the brain into an electromagnetic model creating a chiral image of the sensory input responsible for its creation.

**Figure 11:**
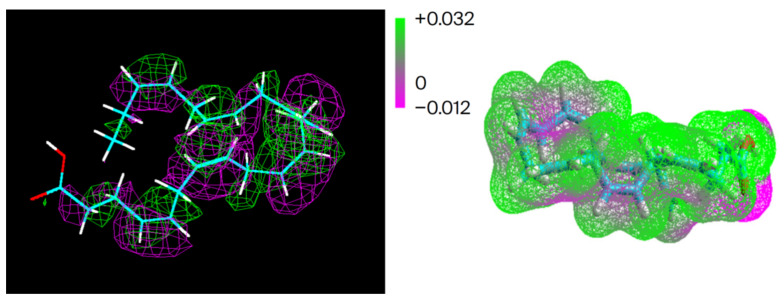
DHA’s electron and spin density (HyperChem 8.0.10 for Windows. Molecular Modeling System Updated 2011, Hypercube, Inc., Gainesville, FL 32601 USA). The mauve is higher energies, and green lower energies. Note that the electron density stretches throughout the length of the molecule. Although all is in motion, there will be a space fill effect portrayed in the righthand image, which also gives an impression of what a memory as a larger, more complex quantum cloud might look like.

DHA has a specific identity, too. In the electron density diagram (Figure 11 below), the green is lower energy, and the mauve higher energy. In the membrane, the electron clouds will lean toward the positive. Moreover, DHA’s uniqueness in stereo-structural, electron, and spin topology is seen in the total spin density of the second image of Figure 11. Adding to DHA’s unique and identifiable contribution to the Hilbert space, Figure 11’s image tells us that the double bonds are not all equal. Their locations within the synaptic map, including electron and spin densities with coordinates and vectors making up a multiple particle field identity in the Hamiltonian of the Hilbert memory space, help paint the electromagnetic shape/identity for a specific memory. 

### 2.5. DHA as a Semiconductor

We propose that DHA’s distinctive six methylene-interrupted cis double bonds confer quantum-mechanical properties essential for neural signaling. Specifically, DHA’s electron spin density, 12 π-electrons, and semiconductive behavior provide a mechanistic bridge from quantum-scale electron behavior to cellular-scale excitability for rapid information transfer, memory encoding, and recognition at timescales beyond those explainable by ion fluxes or protein conformational changes alone. We further argue that DHA-dependent transcription enhances synaptic protein density and the cooperative dynamics of electrons, linking its molecular properties to neuroplasticity.

With the methylene interruption creating a barrier between the double bonds, DHA is a resistor, but only up to a point which would make it a semiconductor. There is more to DHA than fluidity. 

**Figure 12:**
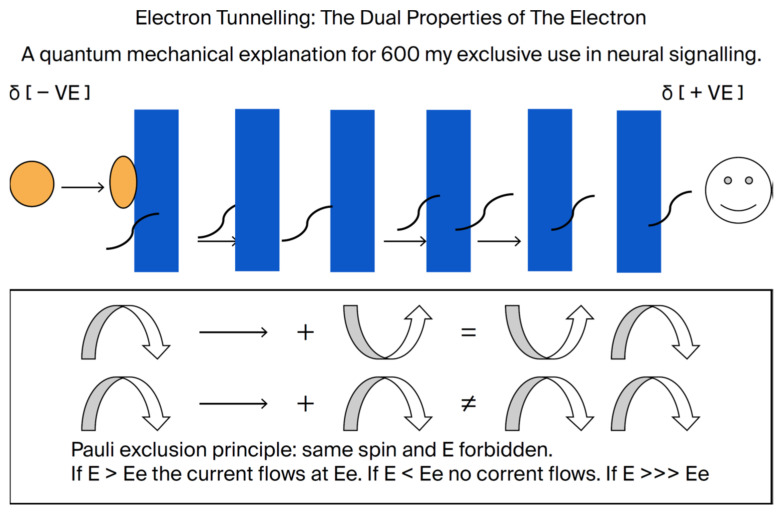
The dual properties of an electron as a particle or a wave provide a plausible explanation for the role of DHA in electrical signaling (constructed by MAC using Word). As demonstrated by our Raman spectroscopy study [33], the methylenes between the double bonds act as barriers to electron flow. If sufficient polarity, as in hyperpolarization, extracts an electron, then another electron can occupy the hole by tunneling through the barriers. However, that electron can only move in if it has the same quantum mechanical properties as the one removed (e.g., energy and spin). Hence, a current can flow with electrons of the same energy and properties, guaranteeing the precision of the signal. As far as we know, there can be no other explanation for the precision of the signals needed for color vision, recognition, memory, recall, and signaling of everyday brain function. The ability to tunnel falls logarithmically with distance. Removal of a double bond makes the distance too far for such tunneling.

**Figure 13:**
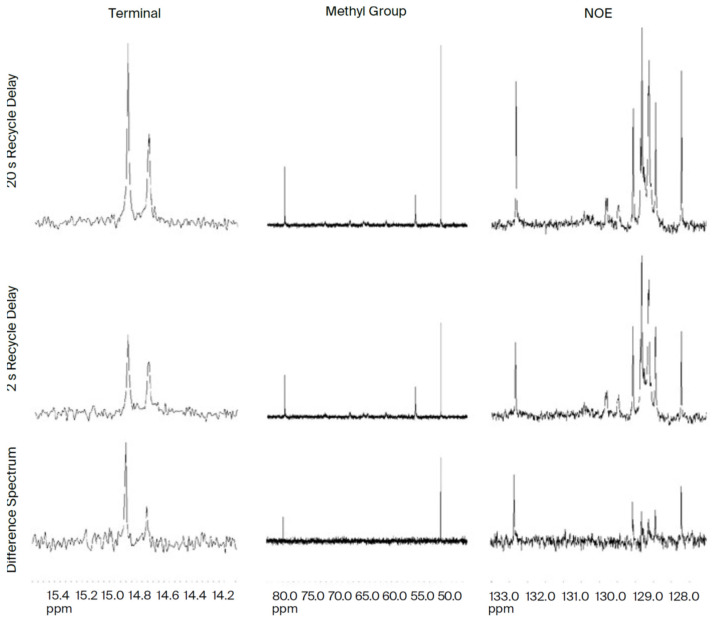
Nuclear Overhauser Enhancement (NOE)**.** The β-chain methyl ppm is more than double by allowing adequate build-up/doubled polarization at the terminal N-NOE, and the α-chain methyl peak at 14.75 ppm is less than the NOE build-up observed at 79.5 ppm. Spectrum is at the central glycerol. Difference does not grow uniformly at the molecular level carbon –CHO-(C=O)-R. NOE polarization build-up occurs at two CH=CH sites at 127.6 ppm and 132.8 ppm. Proton magnetic moment 4 times > 13C.

To obtain evidence of electrical potential in DHA, we used nuclear magnetic resonance (NMR) with Nuclear Overhauser Effect Spectroscopy (NOESY). See Figure 12 and Figure 13 below. Using NOESY, the intrinsic angular momentum of elementary particles is found to align with a given direction. According to the Pauli Exclusion Principle, the spin of a pair of adjacent electrons in an outer shell must have opposite spins (±*ħ*/2.). The Nuclear Overhauser Effect (NOE) is the transfer of nuclear spin polarization from one population of spin-active nuclei (e.g., 1H, 13C, 15N, etc.) to another via cross-relaxation [31] (Figure 12 and Figure 13).

In our NMR study (see Figure 11 above) [31], the head groups, cis double bonds, and terminal methyl group showed up as expected. Further, our calculations based on the Kronig–Penney model asserted that DHA could have at least one conduction band to extract an electron. 

While the methylene interruption of the double bonds in DHA creates a barrier to electron flow, it is probable that the electrochemical gradient across the membrane can become sufficient to extract an electron that penetrates the barrier [14]. Hence, added to the intrinsic dipole, the outer aspect will be positive and the inner aspect negatively maintained by the ionic distribution, in effect providing a cathode and an anode. The resultant hole would then be occupied by another downstream electron, and a current would flow. According to the Pauli Exclusion Principle, the downstream electron can only occupy the hole if it has identical quantum mechanical properties. That is, only electrons of identical quantum properties can flow. This quantum property, known as electron tunneling, provides absolute precision to the signal [32,33], which is essential for color vision and image transfer in the brain [7]. We know of no other explanation for the precision essential to brain function.

We propose that semiconduction by DHA is via quantum tunneling. According to Hopfield, the probability of an electron being found distant from its home falls off logarithmically with the limit approaching zero at 8 Å [32]. This limit is compatible with DHA reaching the terminal omega 3 methyl end at 6 Å. Note in the NOE study (Figure 13 above) that the terminal methyl end of DHA shows an electromagnetic response.

The “push-pull” effect is well known in electrical circuitry as a highly sensitive method to control current flow. Consistent with the experimental establishment of cognitive circuits during gestation, infancy, and learning, that means a high membrane DHA/low arachidonic acid (ARA) state would favor electrical conduction through DHA and a low DHA/high ARA state would do the opposite. 

Molecular dynamic modeling of DHA-rich membrane phosphoglycerides tells us that the double bonds can lie closer together when they are adjacent than they do in the DHA molecule. See Figure 3 above. Accordingly, in the merging of the sensory input and image in memory, the planar structure of DHA adds to the probability, based on the quantum tunneling method [3,7], that there will be sets of two DHAs in parallel halving the resistance leading to the avalanche of current flow and recognition.

The tunneling transmission of current is a special case of operation as a diode. With an anode and cathode across the membrane, there is plausible triode behavior where there is a DHA top and bottom in the bi-layer and a grid in between cathode and anode for the incoming signal. When the incoming wave turns the grid positive, many electrons flow. As the grid turns negative, the flow drops. This effect was used in early radios.

Carbon 6 and silicon 14 are in the same column in the periodic table with only four electrons in the outer shell, which make silicon valuable as a semiconductor. As the nuclei of both carbon and silicon would prefer eight electrons in their outer shells, amplification of the signal from the cohesion of a sensory input and its chiral structure in memory would contribute to the avalanche of electron flow, which results in recognition.

Hui-Min Su of the National Taiwan University College of Medicine, Taipei, proposed a model of the n-3 fatty acids in the development and maintenance of learning memory performance, writing the following: “In these DHA-depleted hippocampi, we observed significantly impaired long-term potentiation (LTP), a well-characterized form of synaptic plasticity similar to that involved in hippocampus-based learning and memory” [34].

### 2.6. Insufficiency of Protein Theory

Although Dr. Su’s paper supports the notion of a central relationship of DHA to memory and cognition, it does not discuss DHA’s electron function and special quantum properties. In general, there has been little attention to the specificity of DHA’s six methylene-interrupted double bonds and their electrical potential by anyone. Rather, the strengthening of synapses by protein was based on repetition of turnover accumulating protein during reconstruction and thereby became the accepted explanation of learning and memory. 

But protein enhancement has another explanation. Hiromitsu Suzuki [35] showed how synapses, including their microsomes and mitochondria, incorporated DHA in preference to similar fatty acids. Bazan et al. [36], described this enhancement of DHA in the turnover of retinal epithelial pigment cells during visual reception. The preferential incorporation of DHA into the developing rat pup brain compared to its parent precursor was demonstrated by Andrew Sinclair in 1972 [37].

This enhancement is a common principle described as biomagnification. During pregnancy, the mother’s plasma phospholipid may contain 4–7% DHA. After crossing various placental and fetal membranes, the proportion may reach 18% in the fetal brain, illustrating its powerful selectivity for DHA [38].

As Kitajka and others showed, DHA in the brain is responsible for activating the transcription of several genes, hence increasing protein synthesis [39]. As described above, the increased turnover during learning results in increased synaptic DHA which would be the mechanism for the increased protein, both contributing to synaptic enhancement and formation of a memory.

### 2.7. Lipid and DHA in Memory, Recall, and Recognition

**Figure 14:**
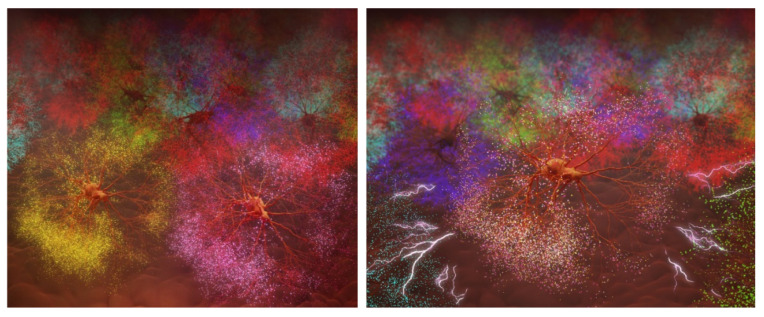
Merger of synaptic cloud with memory. (Artwork by Kevin Heyse, Sage Marketing Group, Denver, CO, using Blender 3D and Photoshop). This is an artist’s impression of the synaptic quantum cloud merging with its corresponding memory. Pre-merger is shown on the **left**, post-merger on the **right**.

We put forward here an electromagnetic hypothesis as to how a memory is mapped in the brain. This is illustrated in Figure 14 below. 

The outer surface of the polar head groups of the synaptic membranes contains a **(+) ve** and **(−) ve** presence due to the choline(+) and phosphorous(−) creating mini-dipoles. These are nano-magnets across plasma membranes distributed throughout the synaptic memory map. In addition, the double bonds in DHA will generate dipoles as previously discussed [3].

A memory will be a three-dimensional map of the dipoles or magnetics as a multiple particle, quantum field. This will be a three-, and possibly four-, dimensional vector field with electron and spin densities adding to the specific shape, geography, and identity of an image received from the sensory system and in memory [40].

The **+ve** and **−ve** must create their own magnetic fields with coordinates and vectors. Taken together, these markers are theoretically capable of forming a quantized, electromagnetic map in three dimensions. Adding the electron and spin density to the scope for quantized imaging is big data on a large scale [40,41]. 

With the creation of a quantized electromagnetic map in three dimensions, we suggest here how the arrangement of membrane dipoles enabled by DHA constructs a memory: 

There will be an extensive arrangement of **+ves** and **−ves**; the flood of electrical information from two retinas will be decoded as a quantum field of synapses; and the plasma membrane of each synapse will contain a set of **+ve−ve+ve−ve** from the choline and phosphorus.

For example, the image of a face will be decoded and possess a geography of **+ves −ves,** e.g.,

**R1+ve−ve+ve−ve−+ve−ve+ve−ve−+ve−ve R2**

In quantum mechanics, this could form a set of wave forms which would create an identity that will become the synaptic cloud in memory.

**Decode**

**R1+ve−ve+ve−ve+ve−ve+ve−ve+ve−ve R2**

**Memory**

**M3−ve+ve−ve+ve−ve+ve−ve+ve−ve+ve M4**

Each **+ve** and **−ve** is a vector which could be part of a quantum field. The magnetic flux between the two sets will continuously create a wave form unless separated by **+ve −ve −ve +ve** or by **−ve +ve +ve −ve** and by non-polar groups such as the rafts, common in synapses, which have a raised density of cholesterol that creates boundaries.

Included in the formation of the imagery is the DHA-enabled dipole across the membranes. The ionized, quaternary amine of choline is matched on the inner surface by ethanolamine, a primary amine which does not ionize under physiological conditions. As the phosphates cancel out, there will be a dipole for outer **+ve** to **−ve** inner membrane leaflets.

The cluster of synapses will be a three-dimensional, multiple-particle system. The topology of the electromagnetic wave forms, and, in particular, the electron spin topology of DHA would create a unique structure of electrical wave form matrices. See Figure 14 below. It takes many iterations to form a chiral image that is the memory of a face. Then, for example, on meeting a person again, the decoded image, entering wave after wave when looking at the face, will search for its chiral image.

The sensory input and its chiral spin wave image are, in effect, Hilbert spaces [40,42,43], each containing a matrix of stereochemical dipoles, which, with electron and spin density topologies, make up a multiple-particle quantum field, the Hamiltonian of which becomes a memory. The electromagnetic pixel-like shapes are spread throughout the memory cloud with multiple coordinates and vectors. These have **(+)ve** and **(−)ves**, each with their own field that together constitute an integrated collection of synapses, comprising a geography or Hilbert space as a quantized identifiable entity. See Figure 14 below.

A paper by Georgiev [44] explains “The most important departure from the deterministic clockwork world of classical physics is the introduction of quantum potentialities and actualities represented by two fundamentally different mathematical objects, namely, state vectors and observable operators on Hilbert space.” The whole world is an electromagnetic shape capable of residency in the brain. 

So much entanglement takes place instantly that it defies classical explanations limited to ion and protein movements. It is likely a function of quantum mechanics with some form of large-scale entanglements and cohesive forces: “the quantum brain”. Hameroff and Sir Roger Penrose fittingly commented that we may need a new physics to explain brain function [45].

### 2.8. Hebbian Learning

The concept of Hebbian learning introduced by Donald Hebb [46] is about the activation of neurons and their connection with other neurons to form a neural network. Each time the stimulus is repeated, the connections grow stronger until the action becomes intuitive:

With Δw representing the change in weight, η the learning rate, and x and y the pre- and post-synaptic activities, respectively. Our discussion, however, focuses not on protein weight gain but rather on π-electron gain, which is facilitated by the 12 π-electrons of DHA. While we do not rule out contribution by other electron paths such as the four double-bond sequence of ARA, in our concept the equation would be

Δπ = ηxy.

where Δ*π* is the difference in electron density, however, this equation does not uncover the mechanism of learning. The storage of information in readable symbolic form is not foreign to the conceptual framework of contemporary biologists. Both DNA and RNA carry information forward, and there is elaborate machinery at the cellular level for reading it [47]. Again, the question of speed arises, and indeed the brain can be trained to speed up responses and recalls [48].

The recording of a name enters the brain via the auditory route and the face from the retina. Within the model of the face, there will be a quantized field or cloud representing the name of the face entangled with the facial model enabled by multi-particle entanglement demanded by relativistic quantum mechanics [49].

Chaos theory is at the heart of our hypothesis [49,50]. Memories are hit by the random/chaotic movement of electrons during sleep, thereby refreshing the membrane, electron density, and protein enhancement, which is LTP. The reactivation of hippocampal memories during sleep has been discussed by Andrew Wikenheiser [51], who proposed that both the orbitofrontal cortex (OFC) and hippocampus have "cognitive maps" that provide useful scaffolds for planning complex behaviors. Touching on different memories during REM sleep could be synthesized into dreams, which are themselves chaotic. 

The same principle of chaotic or random electron flow applies to random thoughts without conscious command or intention. Archimedes’ Eureka moment is a classic example. The brain is never silent.

Electrochemical studies demonstrate that DHA-rich membranes support rapid electron tunneling and exciton migration. Unlike saturated or monounsaturated chains, DHA reduces activation barriers for protein conformational changes, enabling ultrafast kinetics. Its ability to stabilize multiple resonance states allows membranes to function as electron-conducting lattices effectively accelerating signal transduction underlying the efficiency of voltage-gated K^+^ channels and synaptic vesicle cycling.

In this framework, cognition is not solely the sum of protein switches but the emergent behavior of lipid–electron ensembles. Proteins provide specificity and plasticity, while DHA supplies the quantum substrate for the ultrafast matching of patterns.

## 3. Materials and Methods

In this article, we present a theory based on known research that integrates molecular evolution, quantum biology, and membrane dynamics into a unified framework: (1) DHA as a primordial chromophore behind the origin of nervous systems; (2) the electrochemical and quantum properties of DHA in neural membranes; and (3) the role of DHA-mediated electron dynamics in recognition, recall, and plasticity. Together, these considerations point to a new paradigm in which the brain, being largely made of lipids, is understood as a lipid–electron system with DHA at its core. We have searched PUBMED using keywords for relevant papers to integrate with our evidence and with aid of HyperChem, NMR, Nuclear Overhauser Enhancement, Raman spectroscopy, molecular dynamic geometries, quantum mechanical simulation (i.e., self-consistent Density Functional-based Tight Binding (DFTB3)) software, Mulliken–Hush (GMH) method, transition-density couplings via Time-Dependent Density Functional theory (TDDFT), other quantum mechanics tools, Blender 3D, Photoshop, and Hebbian learning—using its direction toward a new theory to explain DHA’s extreme conservation in photoreception, neurons and synapses.

## 4. Conclusions

Docosahexaenoic acid is more than a nutrient or membrane component. It is a molecular invariant that shaped the evolution of nervous systems and continues to support cognition. By functioning as a chromophore-like molecule, an electron conductor, and a quantum substrate, DHA transforms neural membranes into active information-processing participants.

Reframing DHA as the original chromophore and a quantum-active, lipid–electron system in the brain, with proteins providing energy to create a distributed map of magnets representing retrievable memories as three-dimensional quantum clouds generated by dipoles within DHA-rich neuronal membranes, offers a new perspective on cognition. Guided by both classical and quantum principles, recognition, recall, and plasticity emerge from interactions at the membrane–protein interface. Instead of proteins alone, the lipid–electron ensemble is the substrate of thought, and the evolutionary lesson becomes clear: DHA is not a biochemical accident but the molecular foundation of cognition.

## Data Availability

The original contributions presented in this study are included in the article. Further inquiries can be directed to the corresponding author.

## References

[1] Darwin C. (1859). On the Origin of Species by Means of Natural Selection, or the Preservation of Favoured Races in the Struggle for Life.

[2] Kuroha S., Katada Y., Isobe Y., Uchino H., Shishikura K., Nirasawa T., Tsubota K., Negishi K., Kurihara T., Arita M. (2023). Long chain acyl-CoA synthetase 6 facilitates the local distribution of di-docosahexaenoic acid- and ultra-long-chain-PUFA-containing phospholipids in the retina to support normal visual function in mice. FASEB J..

[3] Crawford M.A., Broadhurst C.L., Guest M., Nagar A., Wang Y., Ghebremeskel K., Schmidt W.F. (2013). A quantum theory for the irreplaceable role of docosahexaenoic acid in neural cell signalling throughout evolution. Prostaglandins Leukot. Essent. Fat. Acids.

[4] Kohn W., Sham L.J. (1965). Quantum Density Oscillations in an Inhomogeneous Electron Gas. Phys. Rev..

[5] Car R., Parrinello M. (1985). Unified Approach for Molecular Dynamics and Density-Functional Theory. Phys. Rev. Lett..

[6] Warshel A., Levitt M. (1976). Theoretical studies of enzymic reactions: Dielectric, electrostatic and steric stabilization of the carbonium ion in the reaction of lysozyme. J. Mol. Biol..

[7] Crawford M.A., Sinclair A.J., Wang Y., Schmidt W.F., Broadhurst C.L., Dyall S.C., Horn L., Brenna J.T., Johnson M.R. (2023). Docosahexaenoic Acid Explains the Unexplained in Visual Transduction. Entropy.

[8] Naguib Y.W., Lansakara-P D., Lashinger L.M., Rodriguez B.L., Valdes S., Niu M., Aldayel A.M., Peng L., Hursting S.D., Cui Z. (2016). Synthesis, characterization, and in vitro and in vivo evaluations of 4-(N)-Docosahexaenoyl 2′, 2′-Difluorodeoxycytidine with potent and broad-spectrum antitumor activity. Neoplasia.

[9] Gale M.M., Crawford M.A., Woodford M. (1969). The fatty acid composition of adipose and muscle tissue in domestic and free-living ruminants. Biochem. J..

[10] Anderson R.E., Maude M.B., Zimmerman W. (1975). Lipids of ocular tissues—X. Lipid composition of subcellular fractions of bovine retina. Vision. Res..

[11] Svennerholm L. (1968). Distribution and fatty acid composition of phosphoglycerides in normal human brain. J. Lipid Res..

[12] Svennerholm L., Vanier M.T. (1973). The distribution of lipids in the human nervous system. IV. Fatty acid composition of major sphingolipids of human infant brain. Brain Res..

[13] Crawford M., Marsh D.D.E. (1989). The Driving Force. Chapter 3, The Chemicals of Life.

[14] Crawford M.A., Casperd N.M., Sinclair A.J. (1976). The long chain metabolites of linoleic acid linolenic acids in liver and brain in herbivores and carnivores. Comp. Biochem. Physiol. B.

[15] Crawford M.A., Hassam A.G., Williams G., Whitehouse W.L. (1976). Essential fatty acids and fetal brain growth. Lancet.

[16] Williams G., Crawford M.A. (1987). Comparison of the fatty acid component in structural lipids from dolphins, zebra and giraffe: Possible evolutionary implications. J. Zool. Lond..

[17] Caraveo-Patin J., Wang Y., Soto L.A., Ghebremeskel K., Lehane C., Crawford M.A. (2009). Eco-physiological repercussions of dietary arachidonic acid in cell membranes of active tissues of the Gray whale. Mar. Ecol..

[18] Zhuravlev A., Riding R. (2000). The Ecology of the Cambrian Radiation.

[19] van Dishoeck E.F. (2014). Astrochemistry of dust, ice and gas: Introduction and overview. Faraday Discuss..

[20] Crawford M.A., Schmidt W.F., Broadhurst C.L., Wang Y. (2021). Lipids in the origin of intracellular detail and speciation in the Cambrian epoch and the significance of the last double bond of docosahexaenoic acid in cell signaling. Prostaglandins Leukot. Essent. Fat. Acids.

[21] Zimmerberg J., Gawrisch K. (2006). The physical chemistry of biological membranes. Nat. Chem. Biol..

[22] Crawford M.A., Gale M.M., Woodford M.H. (1969). Linoleic acid and linolenic acid elongation products in muscle tissue of *Sncerus caffer* and other ruminant species. Biochem. J..

[23] Mouritsen O.G. (2011). Model answers to lipid membrane questions. Cold Spring Harb. Perspect. Biol..

[24] McFadden J., Al-Khalili J. (2018). The origins of quantum biology. Proc. R. Soc. A.

[25] Foundation T.N. (2014). The Nobel Prize in Physiology or Medicine 1967. Nobel Media AB. https://www.nobelprize.org/search/?s=George+Wald&nonce=1764295200000.

[26] Carr J.S., Najita J.R. (2008). Organic molecules and water in the planet formation region of young circumstellar disks. Science.

[27] Pakkenberg B., Pelvig D., Marner L., Bundgaard M.J., Gundersen H.J., Nyengaard J.R., Regeur L. (2003). Aging and the human neocortex. Exp. Gerontol..

[28] Watkins E.B., Miller C.E., Majewski J., Kuhl T.L. (2011). Membrane texture induced by specific protein binding and receptor clustering: Active roles for lipids in cellular function. Proc. Natl. Acad. Sci. USA.

[29] Marr D. (1980). Visual information processing: The structure and creation of visual representations. Philos. Trans. R. Soc. Lond. B Biol. Sci..

[30] Siegel A., Sapru H.N. (2006). Essential Neuroscience.

[31] Vögeli B. (2014). The nuclear Overhauser effect from a quantitative perspective. Prog. Nucl. Magn. Reson. Spectrosc..

[32] Hopfield J.J. (1976). On electron transfer. Biophys. J..

[33] Broadhurst C.L., Schmidt W.F., Nguyen J.K., Qin J., Chao K., Aubuchon S.R., Kim M.S. (2017). Continuous gradient temperature Raman spectroscopy and differential scanning calorimetry of N-3DPA and DHA from −100 to 10 degrees C. Chem. Phys. Lipids.

[34] Su H.M. (2010). Mechanisms of n-3 fatty acid-mediated development and maintenance of learning memory performance. J. Nutr. Biochem..

[35] Suzuki H., Manabe S., Wada O., Crawford M.A. (1997). Rapid incorporation of docosahexaenoic acid from dietary sources into brain microsomal, synaptosomal and mitochondrial membranes in adult mice. Int. J. Vitam. Nutr. Res..

[36] Bazan N.G., Gordon W.C., Rodriguez de Turco E.B. (1992). Docosahexaenoic acid uptake and metabolism in photoreceptors: Retinal conservation by an efficient retinal pigment epithelial cell-mediated recycling process. Adv. Exp. Med. Biol..

[37] Sinclair A.J., Crawford M.A. (1972). The incorporation of linolenic aid and docosahexaenoic acid into liver and brain lipids of developing rats. FEBS Lett..

[38] Crawford M.A., Sinclair A.J., Hall B., Ogundipe E., Wang Y., Bitsanis D., Djahanbakhch O.B., Harbige L., Ghebremeskel K., Golfetto I. (2023). The imperative of arachidonic acid in early human development. Prog. Lipid Res..

[39] Kitajka K., Sinclair A.J., Weisinger R.S., Weisinger H.S., Mathai M., Jayasooriya A.P., Halver J.E., Puskas L.G. (2004). Effects of dietary omega-3 polyunsaturated fatty acids on brain gene expression. Proc. Natl. Acad. Sci. USA.

[40] Bruno G., Macetti G., Lo Presti L., Gatti C. (2020). Spin Density Topology. Molecules.

[41] Matta C.F. (2014). Modeling biophysical and biological properties from the characteristics of the molecular electron density, electron localization and delocalization matrices, and the electrostatic potential. J. Comput. Chem..

[42] Zhu T.X., Su M.X., Liu C., Liu Y.P., Wang C.F., Liu P.X., Han Y.J., Zhou Z.Q., Li C.F., Guo G.C. (2024). Integrated spin-wave quantum memory. Natl. Sci. Rev..

[43] Penner A.G., von Oppen F., Zarand G., Zirnbauer M.R. (2021). Hilbert Space Geometry of Random Matrix Eigenstates. Phys. Rev. Lett..

[44] Georgiev D.D. (2020). Quantum information theoretic approach to the mind-brain problem. Prog. Biophys. Mol. Biol..

[45] Hameroff S., Penrose R. (2014). Consciousness in the universe: A review of the ‘Orch OR’ theory. Phys. Life Rev..

[46] Morris R.G. (1999). Do Hebb: The Organization of Behavior, Wiley: New York; 1949. Brain Res Bull..

[47] Gallistel C.R., Balsam P.D. (2014). Time to rethink the neural mechanisms of learning and memory. Neurobiol. Learn. Mem..

[48] Trugenberger C.A. (2001). Probabilistic quantum memories. Phys. Rev. Lett..

[49] Lai Y.C., Xu H.Y., Huang L., Grebogi C. (2018). Relativistic quantum chaos-An emergent interdisciplinary field. Chaos.

[50] Crawford M., Thabet M., Wang Y., Broadhurst C.L., Schmidt W.F. (2018). A theory on the role of π-electrons of docosahexaenoic acid in brain function-The six methylene-interrupted double bonds and the precision of neural signaling. OCL.

[51] Wikenheiser A.M., Gardner M.P.H., Mueller L.E., Schoenbaum G. (2021). Spatial Representations in Rat Orbitofrontal Cortex. J. Neurosci..

